# Serum Prokineticin-2 and Calprotectin as Potential Biomarkers of Disease Stage and Focal Organ Involvement in Human Brucellosis: A Prospective Case–Control Study

**DOI:** 10.3390/diagnostics16203272

**Published:** 2026-10-09

**Authors:** Nazan Cinislioğlu, Nurdan Pür, Murat Daşhan, Nurinnisa Öztürk

**Affiliations:** 1 Department of Infectious Diseases and Clinical Microbiology, Erzurum City Hospital, Erzurum 25240, Türkiye; 2 Department of Medical Biochemistry, Faculty of Medicine, Atatürk University, Erzurum 25240, Türkiye

**Keywords:** biomarkers, brucellosis, calprotectin, focal organ involvement, prokineticin-2

## Abstract

**Background/Objectives:** Brucellosis is a systemic zoonotic infection with a heterogeneous clinical presentation, and early identification of patients at risk for focal organ involvement remains challenging. This study aimed to assess whether serum prokineticin-2 (PROK2) and calprotectin levels are associated with focal organ involvement in patients with brucellosis and to examine their variation according to disease stage. **Methods:** In this prospective case–control study, 150 adults were enrolled, including 90 patients with brucellosis diagnosed based on compatible clinical findings together with a Coombs anti-Brucella titer of ≥1:160 and 60 symptomatic non-brucellosis controls in whom brucellosis was excluded after clinical and laboratory evaluation. The brucellosis group included 30 patients each with acute, subacute, and chronic disease; 50 patients had focal organ involvement and 40 had no focal involvement. Focal organ involvement was defined by organ-specific clinical findings supported by appropriate imaging and/or laboratory methods. Serum PROK2 and calprotectin levels were measured using enzyme-linked immunosorbent assay before antimicrobial treatment, while C-reactive protein (CRP) was evaluated as a routine inflammatory comparator. Comparisons were performed according to disease status, disease stage, and focal organ involvement. Receiver operating characteristic (ROC) curve analysis was used to evaluate discriminative performance for focal organ involvement. A parsimonious multivariable logistic regression model including PROK2, calprotectin, and CRP was used to identify factors independently associated with focal organ involvement. **Results:** Serum PROK2 and calprotectin levels were significantly higher in patients with brucellosis than in the non-brucellosis control group (both *p* < 0.01). PROK2 levels showed significant stage-related variation, with significantly higher adjusted levels in the acute than in the subacute stage, whereas the acute–chronic difference was not statistically significant. Patients with focal organ involvement had significantly higher serum PROK2, calprotectin, and C-reactive protein (CRP) levels than those without focal involvement (all *p* < 0.001). In multivariable analysis, PROK2 (adjusted OR 3.910, 95% CI 1.526–10.018; *p* = 0.005), calprotectin (per 100 ng/mL; adjusted OR 2.223, 95% CI 1.208–4.091; *p* = 0.010), and CRP (per 10 mg/L; adjusted OR 1.662, 95% CI 1.202–2.298; *p* = 0.002) were independently associated with focal organ involvement. For discriminating patients with and without focal organ involvement, the AUCs were 0.877 (95% CI: 0.794–0.959) for calprotectin, 0.864 (95% CI: 0.783–0.946) for CRP, and 0.807 (95% CI: 0.720–0.895) for PROK2. At the exploratory Youden-derived cut-offs, sensitivity/specificity were 92.0%/70.0%, 82.0%/77.5%, and 82.0%/60.0%, respectively. **Conclusions:** Both PROK2 and calprotectin were independently associated with focal organ involvement; however, PROK2 showed stage-related variation and calprotectin was not independently associated with disease stage. Further studies are required to determine their clinical and prognostic value in the assessment of focal organ involvement.

## 1. Introduction

Brucellosis is a systemic zoonotic disease; it is estimated that there are more than two million new cases worldwide each year and it continues to pose a significant public health burden, particularly in endemic regions such as the Mediterranean basin, the Middle East, and Turkey’s Eastern Anatolia Region [1,2,3]. Infections caused by Brucella species can present across a broad clinical spectrum, ranging from acute to chronic disease, and can affect numerous organ systems [4,5]. This clinical heterogeneity complicates diagnostic evaluation and disease management, particularly due to the variable course of focal organ involvement [5,6,7]. Osteoarticular, genitourinary, hepatobiliary, and neurological complications increase disease-related morbidity and may require longer-term or more intensive antimicrobial therapy depending on the site of involvement [4,5,6]. However, while serological tests remain fundamental tools for diagnosis, the role of traditional inflammatory markers such as C-reactive protein (CRP) and erythrocyte sedimentation rate (ESR) in identifying focal organ involvement in human brucellosis remains incompletely defined [7,8,9]. Consequently, there is growing interest in biomarkers that may provide information regarding the host inflammatory response and clinical manifestations of brucellosis [10,11,12].

Although the control of Brucella infection relies primarily on a T helper 1 (Th1)-dominant cellular immune response, components of the innate immune system—particularly the monocyte–macrophage lineage—also play a central role in the early containment of the pathogen and the regulation of the inflammatory response [4,10,13]. Calprotectin (S100A8/S100A9) is a protein of the innate immune system primarily released by activated neutrophils and monocytes; it plays a role in antimicrobial activity, leukocyte migration, and the regulation of the inflammatory response [14,15,16]. Although it has been shown that circulating calprotectin levels may be associated with the presence of infection and inflammatory burden in various bacterial infections, data regarding its clinical value in human brucellosis are quite limited [15,17,18,19].

Prokineticin-2 (PROK2) is a chemokine-like peptide involved in the regulation of hematopoiesis, angiogenesis, and inflammatory cell migration; it plays a role in shaping the innate immune response primarily through myeloid cells, including neutrophils, monocytes, and macrophages [20]. Experimental studies have shown that PROK2 plays a role in the regulation of proinflammatory cytokine production, chemokine signaling, and macrophage-mediated antibacterial defense [20,21]. It has been reported that PROK2 expression can vary in various inflammatory diseases and is associated with the host’s immune response to bacterial infections [20,21,22]. However, these established biological functions do not demonstrate a specific role for PROK2 in human brucellosis. Given the involvement of myeloid cells in the host response to Brucella infection, we hypothesized that PROK2-related inflammatory responses might differ according to disease stage and the presence of focal organ involvement. On this basis, we investigated circulating PROK2 levels across these clinically defined subgroups. To the best of our knowledge, serum PROK2 levels have not previously been evaluated in human brucellosis.

In this prospective case–control study, we aimed to compare serum PROK2 and calprotectin levels between patients with brucellosis and non-brucellosis controls and to assess their associations with disease stage and focal organ involvement.

## 2. Materials and Methods

### 2.1. Study Design and Study Site

This prospective case–control study was carried out between August and December 2025 at the Department of Infectious Diseases and Clinical Microbiology, Erzurum City Hospital, a tertiary referral center serving the Eastern Anatolia region of Türkiye, where brucellosis remains endemic. Consecutive adult patients presenting to the study center during the study period were prospectively screened for eligibility according to predefined inclusion and exclusion criteria, and all eligible patients who provided written informed consent were consecutively enrolled. Ethical approval was granted by the Scientific Research Ethics Committee of Erzurum Faculty of Medicine (Decision No. 2025/07-195). The study was conducted in accordance with the Declaration of Helsinki and approved by the Institutional Ethics Committee. Written informed consent was obtained from all participants prior to their inclusion in the study.

### 2.2. Patient Selection

A total of 150 participants were included in the study. Of these, 90 were patients diagnosed with brucellosis, while 60 formed the control group. A stage-stratified, quota-based consecutive recruitment approach was used for the brucellosis group: within each predefined disease-stage category (acute, subacute, and chronic), eligible patients were consecutively enrolled until the target of 30 patients per stage was reached. The control group consisted of adult cases who were hospitalized and evaluated due to symptoms such as fever, arthralgia, myalgia, and signs of systemic inflammation—which could mimic the clinical presentation of brucellosis—but in whom a diagnosis of brucellosis was ruled out following comprehensive clinical, serological, and microbiological investigations. The control group included cases of bacterial infections, viral infections, cases in which causes of fever other than brucellosis were investigated, and non-infectious inflammatory conditions. In patients classified as having undiagnosed febrile illness, brucellosis was specifically excluded through clinical assessment and brucellosis-specific serological testing, with microbiological investigations performed where clinically indicated. The term ‘undiagnosed febrile illness’ referred to patients in whom no definitive alternative cause of fever was identified despite diagnostic evaluation, rather than to uncertainty regarding the exclusion of brucellosis. The exclusion criteria referring to autoimmune/rheumatologic diseases, inflammatory bowel disease, and other active chronic inflammatory diseases were intended to exclude patients with pre-existing chronic or persistently active inflammatory disorders that could independently influence circulating inflammatory biomarker levels. In contrast, the non-infectious inflammatory control subgroup consisted of patients presenting with acute inflammatory conditions who had no previously known chronic active inflammatory disease and in whom brucellosis was excluded during the index diagnostic evaluation. The detailed diagnostic distribution of the control group is presented in the results section. A healthy control group was not included because the primary aim of the control strategy was to compare patients with brucellosis with symptomatic patients presenting with clinically relevant infectious or inflammatory conditions in whom brucellosis had been excluded, rather than with individuals without an inflammatory response. Given that both PROK2 and calprotectin are inflammation-related biomarkers, comparison with such clinical controls was considered more informative for assessing whether their circulating levels were associated with brucellosis beyond a nonspecific inflammatory response.

Diabetes mellitus, hypertension, and stable coronary artery disease were not considered exclusion criteria. However, to minimize potential confounding factors that could affect serum calprotectin and PROK2 levels, active malignancy, advanced-stage chronic kidney disease (stages 4–5), chronic liver failure, autoimmune diseases, rheumatological diseases, inflammatory bowel disease, active chronic inflammatory disease, concurrent systemic infection, primary or secondary immunodeficiency, use of immunosuppressive therapy, pregnancy and cases with incomplete clinical or laboratory data, including patients in whom symptom duration could not be determined with sufficient reliability for disease-stage classification, were excluded from the study. The screening, inclusion, and exclusion criteria for patients evaluated during the study period are summarized in a flowchart in accordance with STROBE guidelines, and the relevant numbers are presented in the Results Section.

### 2.3. Diagnosis, Classification, and Treatment Approach for Brucellosis

Brucellosis was diagnosed in patients with compatible clinical findings and a Coombs anti-Brucella titer of ≥1:160, which constituted the common serological criterion for all included cases; additional serological findings and microbiological confirmation were considered supportive when available [23,24]. As part of the predefined diagnostic work-up, Rose Bengal, STA, Coombs anti-Brucella, and Brucella-specific IgM and IgG tests were performed in all patients with brucellosis. Serological findings were interpreted in conjunction with compatible clinical findings and diagnostic titers and/or microbiological evidence. In our diagnostic algorithm, a Coombs anti-Brucella titer of ≥1:160 was used as the common serological criterion. A standard tube agglutination (STA) titer of ≥1:160 provided additional serological support but was not required when the Coombs anti-Brucella test was positive. Isolation of Brucella spp. from blood or an appropriate clinical specimen was considered definitive microbiological confirmation; however, culture positivity was not mandatory when compatible clinical findings were accompanied by positive serology [24].

Patients were categorized according to symptom duration as having acute (<8 weeks), subacute (8–52 weeks), or chronic (>52 weeks) brucellosis [25]. Symptom onset was defined as the reported onset of the first symptom considered compatible with brucellosis and was determined through a detailed clinical history. Patient-reported symptom duration was corroborated, whenever available, using previous medical records and documented healthcare encounters. When an approximate symptom duration clearly fell within a single disease-stage category, that category was assigned; however, when the reported duration overlapped a stage-defining boundary and could not be reliably resolved using available medical records, the patient was excluded to minimize disease-stage misclassification. Focal organ involvement was defined as organ-specific involvement confirmed by appropriate imaging and/or microbiological methods in the presence of consistent clinical findings [23]. Treatment was initiated after completion of the baseline assessments and was selected according to current guideline recommendations, taking into account disease stage and focal organ involvement [4].

### 2.4. Definition and Classification of Focal Organ Involvement

Focal organ involvement was defined based on consistent clinical findings specific to the relevant organ system, supported by appropriate imaging and/or laboratory methods, consultation with relevant specialties when necessary, and the exclusion of alternative etiologies [7,26]. Osteoarticular involvement was classified as peripheral arthritis and axial involvement (spondylodiscitis and/or sacroiliitis) based on anatomical localization. Peripheral arthritis was defined by the presence of inflammatory clinical findings in peripheral joints, confirmed as needed by ultrasound and/or magnetic resonance imaging (MRI); axial involvement was defined by clinical findings consistent with brucellosis, supported by brucellosis-specific MRI findings [27].

Genitourinary involvement was defined by the confirmation of clinical findings consistent with epididymo-orchitis via scrotal Doppler ultrasound; hepatobiliary involvement was defined in patients with confirmed brucellosis by newly detected elevation in serum aminotransferases (AST and/or ALT) to more than two times the laboratory upper limit of normal and/or cholestatic liver enzymes (ALP and/or GGT) above the laboratory upper limit of normal, in the absence of a more likely alternative cause. Hepatobiliary ultrasonography was used as a supportive assessment, and findings such as hepatomegaly, increased hepatic echogenicity, parenchymal heterogeneity, and/or biliary abnormalities were recorded; imaging findings alone were not considered sufficient to establish hepatobiliary involvement. Viral hepatitis, autoimmune hepatitis, drug-induced liver injury, and other clinically relevant causes of hepatitis were excluded on the basis of clinical history, medication exposure, laboratory evaluation, and imaging findings, as appropriate. Liver enzyme abnormalities were attributed to brucellosis only after these alternative causes had been excluded [26]. The diagnosis of neurobrucellosis was made based on consistent neurological findings, in addition to cerebrospinal fluid analysis and/or findings from cranial and spinal MRI [5,28].

Focal organ involvement was assessed at the patient level as a binary variable (present or absent). Patients with at least one focal organ involvement were classified as having focal organ involvement. In patients with involvement of more than one anatomical site, the patient was counted once in the patient-level analysis, whereas each involved anatomical site was recorded separately for organ-specific descriptive analyses. Therefore, the total number of focal sites could exceed the number of patients with focal organ involvement.

### 2.5. Evaluation of Clinical Data and Routine Laboratory Parameters

Demographic characteristics, comorbidities, duration of symptoms, presence of fever, focal organ involvement, and treatment data were prospectively recorded using standard data collection forms. All clinical and laboratory data were collected based on baseline assessments obtained before the initiation of antibiotic therapy.

Complete blood count, neutrophil and lymphocyte counts, hemoglobin, platelet count, CRP, ESR, and routine biochemical parameters were evaluated from peripheral venous blood samples collected at the time of presentation. CRP was measured at the time of presentation as part of routine clinical laboratory testing using a nephelometric method on a Siemens BN II analyzer (Siemens Healthineers, Marburg, Germany). Results were reported in mg/L, with a laboratory reference range of 0–5 mg/L. CRP was included as an established routine inflammatory comparator to assess the performance of PROK2 and calprotectin relative to a marker commonly used in clinical practice. In patients with brucellosis, Brucella-specific immunoglobulin M (IgM) and immunoglobulin G (IgG) levels were also recorded using the STA and Coombs anti-Brucella test.

### 2.6. Serum Calprotectin and Prok2 Measurements

Peripheral venous blood samples were collected from all participants prior to the initiation of antimicrobial therapy to determine serum calprotectin and PROK2 levels. After allowing the blood samples to clot at room temperature, the serum phase was separated by centrifugation at 3000 rpm for 10 min. Serum samples were aliquoted and stored at −80 °C until analysis, and repeated freeze–thaw cycles were avoided. All samples were analyzed simultaneously during the same analysis period.

Serum calprotectin levels were measured using a commercial human calprotectin sandwich ELISA kit (BT Laboratory, Cat. No: E4010Hu, Shanghai, China), while serum PROK2 levels were measured using a commercial human PROK2 sandwich ELISA kit (Feiyue Biotechnology Co., Ltd., Cat. No: FY-EH3285, Wuhan, China), in accordance with the manufacturers’ protocols. The calprotectin ELISA kit had a measurement range of 2–600 ng/mL and an analytical sensitivity of 1.67 ng/mL, while the PROK2 ELISA kit had a measurement range of 0.32–20 ng/mL and an analytical sensitivity of 0.19 ng/mL.

**Figure 1:**
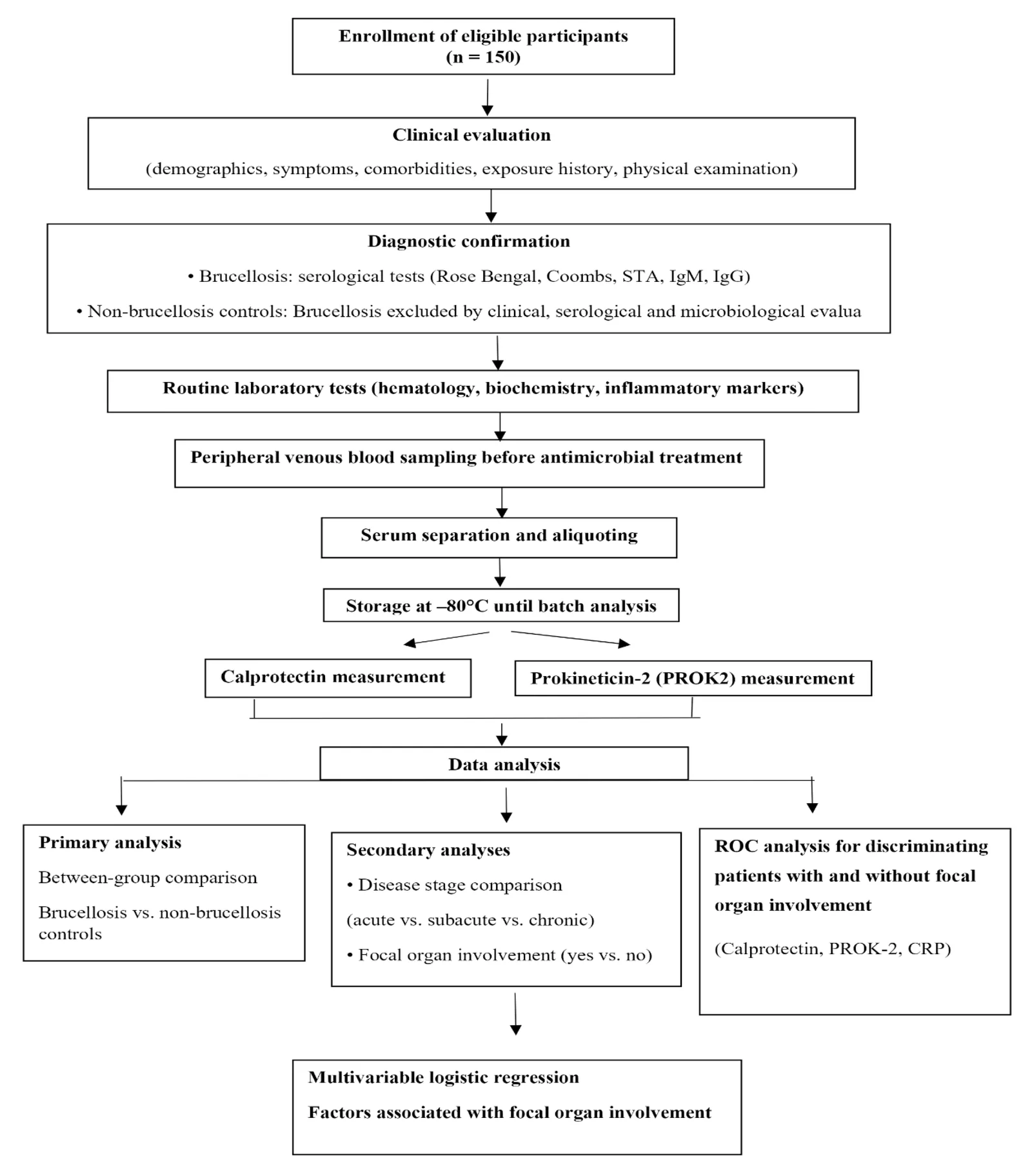
Study workflow, laboratory procedures, and analytical framework.

All samples were analyzed in duplicate, and absorbance values were measured at a wavelength of 450 nm using a microplate reader. Analyte concentrations were calculated using standard curves recommended by the manufacturer. All ELISA analyses were performed by laboratory personnel who were blinded to the patients’ clinical data. The manufacturer reported that the intra-assay coefficient of variation for the calprotectin ELISA kit was <6% and the inter-assay coefficient of variation was <10%; for the PROK2 ELISA kit, both the intra-assay and inter-assay coefficients of variation were <10%. The general workflow of the study, laboratory procedures, and statistical analysis plan are summarized in Figure 1.

### 2.7. Sample Size

Sample size estimation was performed before study initiation using G*Power software (version 3.1.9.7, Düsseldorf, Germany). The calculation was based on the effect size reported in a previous investigation of serum calprotectin in bacterial infections [29]. Assuming a Cohen’s *d* of 0.62, a two-sided significance level of 0.05, and 80% statistical power, the minimum required sample size was estimated to be 78 participants [29]. The calculated sample size represented the minimum required sample rather than a prespecified case-to-control allocation ratio; the final sample exceeded this minimum and comprised 90 patients with brucellosis and 60 non-brucellosis controls. Since there were no prior data on serum PROK2 levels in human brucellosis, PROK2 analyses were planned on an exploratory basis. Because no previous study had specifically evaluated serum calprotectin in human brucellosis, the effect size used for the prespecified sample-size calculation was derived from a previous study evaluating serum calprotectin in bacterial infection [29], which represented the closest available evidence at the time of study planning. This calculation was used to determine the minimum overall sample size for the biomarker comparison. Analyses according to disease stage and focal organ involvement were not the basis of the original formal sample-size calculation. No separate a priori sample-size calculation was performed specifically for the multivariable logistic regression or ROC analyses; these analyses were therefore considered exploratory.

### 2.8. Statistical Analysis

Statistical analyses were carried out using IBM SPSS Statistics for Windows (version 26.0; IBM Corp., Armonk, NY, USA). The distribution of continuous variables was evaluated with the Shapiro–Wilk test. Variables showing a normal distribution are reported as mean ± standard deviation (SD), whereas non-normally distributed variables are expressed as median and interquartile range (IQR). Comparisons among the acute, subacute, and chronic disease groups were performed using one-way analysis of variance (ANOVA) for normally distributed variables and the Kruskal–Wallis test for variables that did not meet the assumption of normality. Homogeneity of variance was examined using Levene’s test. When equal variances were confirmed, Tukey’s Honestly Significant Difference (HSD) test was applied for post hoc analyses; otherwise, the Games–Howell procedure was used. Significant Kruskal–Wallis results were followed by pairwise Mann–Whitney U tests with Bonferroni adjustment. Bonferroni adjustment was applied to post hoc pairwise comparisons following significant overall group tests. No global multiplicity adjustment was applied across all clinical variables and biomarkers because these analyses were considered secondary and exploratory.

Categorical data are presented as frequencies and percentages and were analyzed using either Pearson’s chi-square test or Fisher’s exact test, as appropriate. To evaluate the independent association of PROK2 and calprotectin levels with disease stage, generalized linear models with a gamma distribution and log-link function were performed because both biomarkers showed non-normal distributions. Disease stage was included as a categorical factor, while age, hemoglobin, and CRP were included as covariates. Covariates for the disease-stage models were selected based on clinical relevance together with observed differences across disease-stage groups. Age was included because it differed across disease stages and may influence inflammatory biomarker concentrations. CRP was included as a marker of systemic inflammatory burden that could confound the association between disease stage and biomarker levels, while hemoglobin was included because it also showed significant variation across disease stages and was considered a potential indicator of differences in the patients’ systemic clinical and inflammatory profile. Given the sample size within each disease-stage group, the number of covariates was deliberately limited to reduce model overparameterization. Adjusted estimated means with 95% confidence intervals were calculated, and pairwise comparisons between disease stages were performed using Bonferroni correction.

The ability of serum PROK2, calprotectin, and CRP to discriminate between patients with and without focal organ involvement was assessed by receiver operating characteristic (ROC) curve analysis. Discriminative performance was summarized using the area under the curve (AUC) with corresponding 95% confidence intervals (95% CI), while exploratory cut-off values were derived using the Youden index. Sensitivity and specificity were calculated for each Youden-derived cut-off value.

Factors associated with focal organ involvement were initially explored using univariate binary logistic regression. Variables considered in the analyses were grouped as demographic and clinical characteristics, routine laboratory parameters, and study biomarkers. Epidemiological variables such as residence, occupation, and exposure history were included in analyses of focal organ involvement because of their potential clinical relevance to disease phenotype, whereas disease-stage comparisons focused on demographic, clinical, laboratory, serological, and biomarker variables directly related to the study objectives. Demographic and clinical covariates included sex, residence, and comorbidities; routine laboratory parameters included white blood cell count, platelet count, lymphocyte count, lactate dehydrogenase, erythrocyte sedimentation rate, and C-reactive protein; and PROK2 and calprotectin were evaluated as study biomarkers. A parsimonious multivariable binary logistic regression model including PROK2, calprotectin, and CRP was subsequently constructed. This model was specified a priori for the revised analysis to focus on the two principal study biomarkers and CRP as the established routine inflammatory comparator, while limiting the number of predictors relative to the available number of focal organ involvement events. Accordingly, adjusted odds ratios for continuous biomarkers represent the change in odds associated with prespecified clinically interpretable increments: 1 ng/mL for PROK2, 100 ng/mL for calprotectin, and 10 mg/L for CRP. For categorical variables included in the univariable regression analyses, female sex, urban residence, and absence of hypertension were used as the reference categories. Adjusted odds ratios (ORs) with 95% confidence intervals were reported. Model calibration was evaluated using the Hosmer–Lemeshow goodness-of-fit test. Multicollinearity among the variables included in the final multivariable model was assessed using variance inflation factors (VIFs), with all VIF values < 2.

All statistical analyses were two-tailed, and statistical significance was defined as a *p* value < 0.05.

## 3. Results

**Figure 2:**
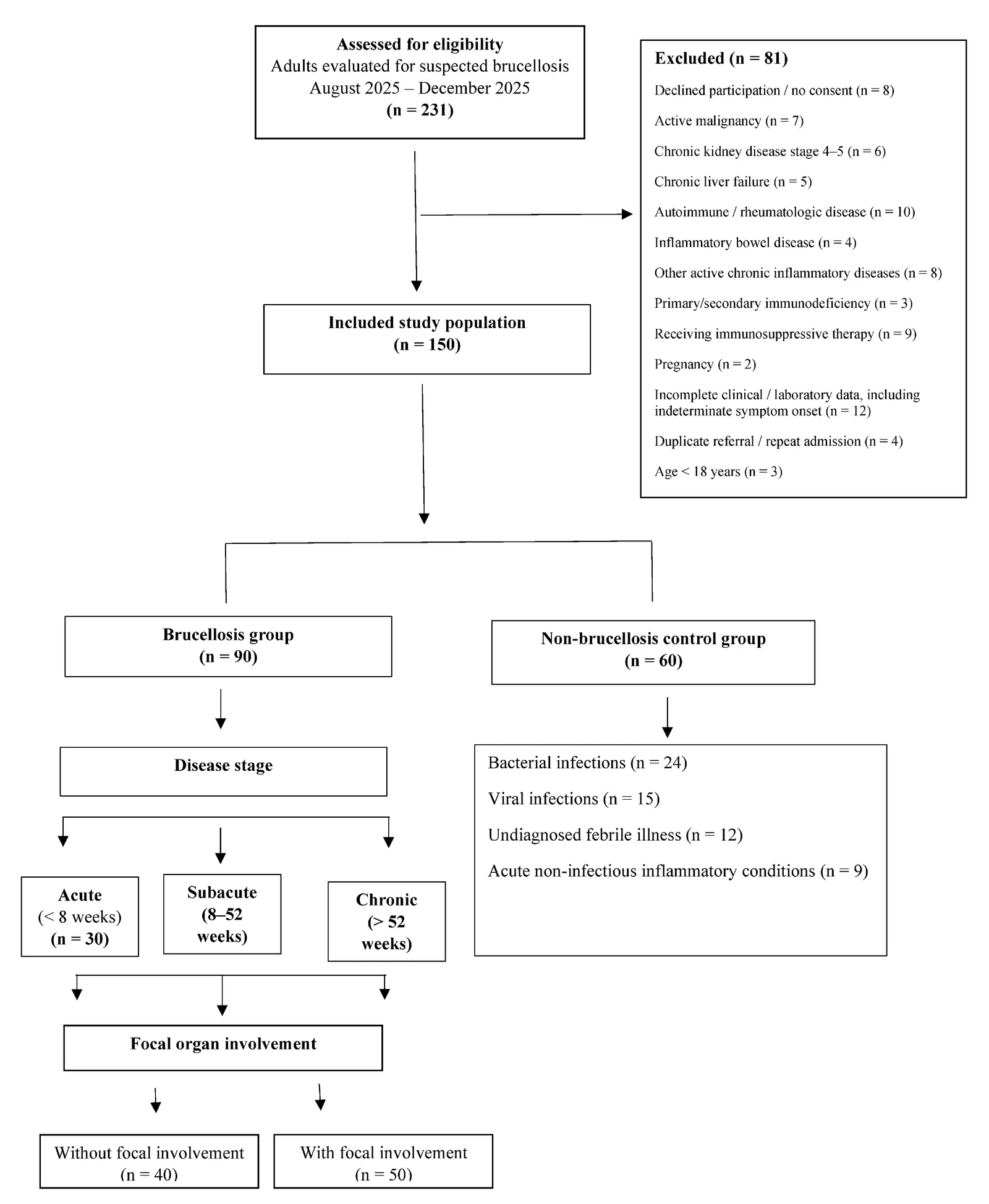
Flow diagram of participant selection and study group allocation.

A total of 231 adult cases evaluated with a preliminary diagnosis of brucellosis during the study period were screened for eligibility. Eighty-one cases (35.1%) were excluded from the study due to exclusion criteria, while 150 participants (64.9%) were included in the study. The study population consisted of 90 brucellosis patients (60.0%) and 60 non-brucellosis control subjects (40.0%). In accordance with the predefined stage-stratified recruitment strategy, 30 patients were enrolled in each disease-stage group—acute, subacute, and chronic (33.3% each); therefore, this equal distribution reflects the recruitment design and should not be interpreted as the natural distribution of disease stages among patients presenting during the study period. Rose Bengal, STA, Coombs anti-Brucella, and Brucella-specific IgM and IgG results were available for all 90 patients with brucellosis. Rose Bengal was positive in 72 patients (80.0%), Brucella-specific IgM in 17 patients (18.9%), and Brucella-specific IgG in 76 patients (84.4%). All 90 patients had a Coombs anti-Brucella titer of ≥1:160. An STA titer of ≥1:160 was present in 18 patients (20.0%), and blood cultures were positive for Brucella spp. in 8 patients (8.9%). Among the eight culture-positive patients, five also had an STA titer of ≥1:160. Focal organ involvement was detected in 50 patients (55.6%) in the brucellosis group, while 40 patients (44.4%) showed no focal organ involvement. Focal organ involvement was present in 15 of 30 patients (50.0%) with acute brucellosis, 16 of 30 (53.3%) with subacute brucellosis, and 19 of 30 (63.3%) with chronic brucellosis, with no statistically significant difference among the three disease-stage groups (*p* = 0.557). Among the 50 patients with focal organ involvement, 26 (52.0%) had peripheral arthritis, 22 (44.0%) had spondylodiscitis/sacroiliitis, 10 (20.0%) had orchitis, 5 (10.0%) had hepatitis, 1 (2.0%) had central nervous system involvement and 1 (2.0%) had pneumonia. Because some patients had involvement at more than one anatomical site, the total number of focal sites was 65; therefore, the sum of the organ-specific involvements exceeded the number of patients with focal organ involvement. The process of participant selection and study group formation is shown in Figure 2.

No statistically significant differences were found between the brucellosis and non-brucellosis control groups in terms of age, gender distribution, presence of fever, and the prevalence of comorbidities (all *p* > 0.05). Similarly, no significant differences were observed between the groups in terms of white blood cell, neutrophil, and lymphocyte counts, as well as hemoglobin, platelet, creatinine, and ESR levels (all *p* > 0.05). Serum CRP levels were found to be statistically significantly higher in the brucellosis group compared to the control group (19.0 [3.1–63.3] mg/L vs. 3.7 [3.1–33.7] mg/L; *p* = 0.001). Similarly, serum PROK2 levels (2.51 [1.57–3.31] ng/mL vs. 2.00 [1.04–2.72] ng/mL; *p* = 0.004) and calprotectin levels (207.9 [71.0–334.5] ng/mL vs. 91.6 [62.9–142.0] ng/mL; *p* = 0.001) were also found to be statistically significantly higher in the brucellosis group. The nine patients classified as having acute non-infectious inflammatory conditions included post-infectious reactive arthritis (n = 2), acute gout (n = 2), chondrocalcinosis/pseudogout (n = 1), drug-induced fever (n = 3), and acute pericarditis (n = 1). The bacterial infection subgroup included complicated urinary tract infection/pyelonephritis (n = 6), community-acquired pneumonia (n = 5), skin and soft tissue infection (n = 5), bacterial gastroenteritis (n = 3), sepsis/bacteremia (n = 3), bacterial meningitis (n = 1), and diabetic foot infection (n = 1), whereas the viral infection subgroup included influenza (n = 5), viral gastroenteritis (n = 4), acute viral hepatitis (n = 3), herpes zoster (n = 2), and viral meningitis/encephalitis (n = 1). A comparison of selected demographic, clinical, and laboratory characteristics between the brucellosis and non-brucellosis control groups is presented in Table 1.

The median serum PROK2 level was higher in the acute group at 3.17 (IQR: 2.27–5.13) ng/mL compared with the subacute and chronic groups, with corresponding median levels of 2.25 (IQR: 1.62–2.87) and 2.07 (IQR: 1.37–2.89) ng/mL, respectively (*p* = 0.015). Furthermore, while statistically significant differences were observed between disease stages in terms of age, white blood cell count, hemoglobin level, lymphocyte count, erythrocyte sedimentation rate, CRP level, Brucella IgM positivity, and Coombs tube agglutination titers (all *p* < 0.05), no significant differences were observed in other clinical, laboratory, and serological parameters (all *p* > 0.05). Rose Bengal positivity and blood culture positivity did not differ significantly across the acute, subacute, and chronic disease-stage groups (*p* = 0.535 and *p* = 0.522, respectively). A comparison of selected demographic, clinical, laboratory, and serological characteristics according to disease stages is summarized in Table 2.

In laboratory analyses, the median serum PROK2 level was 3.17 (IQR: 2.27–5.13) ng/mL in the acute group, which was significantly higher than in the subacute group (2.25 [IQR: 1.62–2.87] ng/mL; *p* = 0.012) and the chronic group (2.07 [IQR: 1.37–2.89] ng/mL; *p* = 0.014). In contrast, no significant differences were detected in serum calprotectin levels in pairwise group comparisons. Furthermore, significant pairwise group differences were identified for leukocytes, lymphocytes, hemoglobin, CRP, and ESR (all *p* < 0.05). The results of Bonferroni-corrected pairwise comparisons are presented in Table 3.

After adjustment for age, hemoglobin, and CRP, disease stage remained significantly associated with PROK2 levels (Wald χ^2^ = 11.039, *p* = 0.004). The adjusted PROK2 and calprotectin levels according to disease stage are presented in Table 4. Adjusted mean PROK2 levels were 3.30 (95% CI 2.73–4.00), 2.13 (1.77–2.56), and 2.33 (1.91–2.83) in the acute, subacute, and chronic groups, respectively. Bonferroni-adjusted analysis showed a significant difference between the acute and subacute groups (*p* = 0.006), while the other pairwise comparisons were not significant.

In contrast, disease stage was not independently associated with calprotectin levels after adjustment (Wald χ^2^ = 3.427, *p* = 0.180), and no significant pairwise differences were observed. In a comparison of brucellosis patients with and without focal organ involvement, male gender (80.0% vs. 47.5%; *p* = 0.001), living in a rural area (80.0% vs. 55.0%; *p* = 0.013), and the presence of hypertension (44.0% vs. 22.5%; *p* = 0.033) were significantly more common in patients with focal organ involvement. Among clinical findings, only low back pain showed a significantly higher prevalence in the group with focal organ involvement (68.0% vs. 40.0%; *p* = 0.015). In laboratory evaluations, white blood cell count, lactate dehydrogenase (LDH), ESR, and CRP levels were statistically significantly higher in patients with focal organ involvement, while platelet and lymphocyte counts were significantly lower (all *p* < 0.05). The median serum PROK2 level was 2.9 (IQR: 2.3–4.3) ng/mL in patients with focal organ involvement and 1.7 (IQR: 1.2–2.5) ng/mL in those without focal organ involvement, and was significantly higher in the focal involvement group (*p* < 0.001). Similarly, the median serum calprotectin level was significantly higher in patients with focal organ involvement than in those without focal organ involvement (319.7 [IQR: 232.6–341.5] ng/mL vs. 69.6 [IQR: 44.6–158.4] ng/mL; *p* < 0.001). In the microbiological evaluation, blood culture positivity was more frequent in patients with focal organ involvement (16.0% vs. 0%; *p* = 0.008). A comparison of demographic, clinical, laboratory, microbiological, and treatment characteristics according to focal organ involvement is presented in Table 5.

**Table 4:** Adjusted PROK2 and calprotectin levels according to disease stage.

Biomarker	Disease Stage	Adjusted Mean	95% CI	Overall *p* *	Pairwise *p* †
**PROK2**	Acute	**3.30**	2.73–4.00	**0.004**	Acute vs. Subacute: **0.006**
	Subacute	**2.13**	1.77–2.56		Acute vs. Chronic: 0.062
	Chronic	**2.33**	1.91–2.83		Subacute vs. Chronic: 1.000
**Calprotectin**	Acute	201.19	155.68–259.99	0.180	Acute vs. Subacute: 0.817
	Subacute	163.49	126.86–210.69		Acute vs. Chronic: 1.000
	Chronic	231.67	178.75–300.25		Subacute vs. Chronic: 0.224

* Overall *p* values were obtained from Type III Wald tests for disease stage. † Bonferroni-adjusted pairwise comparisons. Adjusted estimated means were obtained from generalized linear models with gamma distribution and log-link function, adjusted for age, hemoglobin, and CRP.

In univariate logistic regression analysis, male gender, living in a rural area, white blood cell count, platelet count, lymphocyte count, LDH, erythrocyte sedimentation rate, CRP level, serum calprotectin level, serum PROK2 level, and the presence of hypertension showed a significant association with focal organ involvement (all *p* < 0.05). In multivariable logistic regression analysis, serum PROK2 level (adjusted OR: 3.910; 95% CI: 1.526–10.018; *p* = 0.005), serum calprotectin level (per 100 ng/mL; adjusted OR: 2.223; 95% CI: 1.208–4.091; *p* = 0.010), and CRP (per 10 mg/L; adjusted OR: 1.662; 95% CI: 1.202–2.298; *p* = 0.002) were independently associated with focal organ involvement. Accordingly, each 1 ng/mL increase in PROK2 was associated with a 3.910-fold increase in the odds of focal organ involvement, each 100 ng/mL increase in calprotectin with a 2.223-fold increase in the odds, and each 10 mg/L increase in CRP with a 1.662-fold increase in the odds. The results of univariable and multivariable logistic regression analyses are presented in Table 6. 

**Table 5:** Comparison of demographic, clinical, laboratory, and microbiological characteristics according to focal organ involvement.

Variable	Without Focal Organ Involvement (n = 40)	With Focal Organ Involvement (n = 50)	*p* Value
**Demographic characteristics**			
Age (years), mean ± SD	47.7 ± 11.7	47.0 ± 13.2	0.806 ^b^
Sex, n (%)			**0.001 ^a^**
Female, n (%)	21 (52.5)	10 (20.0)	
Male, n (%)	19 (47.5)	40 (80.0)	
**Comorbidities, n (%)**			
Hypertension	9 (22.5)	22 (44.0)	**0.033 ^a^**
Diabetes mellitus	4 (10.0)	11 (22.0)	0.129 ^a^
Congestive heart failure	2 (5.0)	1 (2.0)	0.583 ^b^
Coronary artery disease	8 (20.0)	10 (20.0)	1.000 ^a^
**Residence, n (%)**			**0.013 ^a^**
Rural	22 (55.0)	40 (80.0)	
Urban	18 (45.0)	10 (20.0)	
**Occupation, n (%)**			**0.005 ^b^**
Farmer	5 (12.5)	6 (12.0)	
Other agricultural occupation	6 (15.0)	22 (44.0)	
Livestock breeder	9 (22.5)	11 (22.0)	
Veterinarian	3 (7.5)	5 (10.0)	
Laboratory worker	0 (0.0)	0 (0.0)	
Other occupations *	17 (42.5)	6 (12.0)	
**Exposure history, n (%)**			0.064 ^b^
Animal contact	0 (0.0)	2 (4.0)	
Animal contact + unpasteurized dairy products	16 (40.0)	30 (60.0)	
Animal contact + occupational exposure	1 (2.5)	0 (0.0)	
Unpasteurized dairy products	14 (35.0)	10 (20.0)	
Occupational exposure	2 (5.0)	5 (10.0)	
Unknown	7 (17.5)	3 (6.0)	
**Clinical characteristics, n (%)**			
Fever	21 (52.5)	24 (48.0)	0.671 ^a^
Weight loss	8 (20.0)	19 (38.0)	0.105 ^a^
Loss of appetite	27 (67.5)	32 (64.0)	0.380 ^a^
Sweating	32 (80.0)	36 (72.0)	0.237 ^a^
Gastrointestinal symptoms	8 (20.0)	16 (32.0)	0.749 ^b^
Fatigue	36 (90.0)	43 (86.0)	0.521 ^b^
Depression	0 (0.0)	3 (6.0)	0.339 ^a^
Arthralgia	27 (67.5)	39 (78.0)	0.781 ^a^
Skin rash	6 (15.0)	9 (18.0)	0.317 ^a^
Headache	9 (22.5)	16 (32.0)	0.445 ^a^
Low back pain	16 (40.0)	34 (68.0)	**0.015 ^b^**
**Laboratory findings**			
WBC (/µL), median (IQR)	6290 (5600–7420)	8480 (6270–9750)	**0.002 ^c^**
Hemoglobin (g/dL)	14.5 ± 1.5	14.8 ± 1.6	0.427 ^b^
Platelet count (/µL), mean ± SD	273,300 ± 69,789.5	237,020 ± 66,513.3	**0.014 ^b^**
Neutrophil count (/µL), median (IQR)	3620 (3195–4550)	4110 (2700–5390)	0.773 ^c^
Lymphocyte count (/µL), mean ± SD	2285.5 ± 613.8	1934.2 ± 670.9	**0.012 ^b^**
Creatinine (mg/dL), mean ± SD	0.77 ± 0.13	0.81 ± 0.15	0.241 ^b^
Albumin (g/L), median (IQR)	43 (41–45)	42 (40–45)	0.323 ^c^
ALT (U/L), median (IQR)	30 (19–45)	29.5 (23–64)	0.235 ^c^
AST (U/L), median (IQR)	20.5 (17–30)	22 (17–42)	0.614 ^c^
LDH (U/L), median (IQR)	186 (176–221)	234.5 (191–286)	**0.023 ^c^**
GGT (U/L), median (IQR)	35 (18.5–65)	30.5 (23–54)	0.807 ^c^
ALP (U/L), median (IQR)	83.5 (56–95.5)	85 (64–110)	0.411 ^c^
ESR (mm/h), median (IQR)	8 (2.5–15)	16.5 (4–35)	**0.013 ^c^**
Ferritin (ng/mL), median (IQR)	68.5 (46.5–102)	89.5 (44.4–144)	0.236 ^c^
CRP (mg/L), median (IQR)	5.4 (3.1–13.0)	63.1 (36.0–83.1)	**<0.001 ^c^**
Prokineticin-2 (ng/mL), median (IQR)	1.7 (1.2–2.5)	2.9 (2.3–4.3)	**<0.001 ^c^**
Calprotectin (ng/mL), median (IQR)	69.6 (44.6–158.4)	319.7 (232.6–341.5)	**<0.001 ^c^**
**Microbiological and serological findings**			
**Blood culture, n (%)**			**0.008 ^b^**
Negative	40 (100.0)	42 (84.0)	
Positive	0 (0.0)	8 (16.0)	
**Rose Bengal, n (%)**			0.112 ^a^
Negative	5 (12.5)	13 (26.0)	
Positive	35 (87.5)	37 (74.0)	
**Brucella IgM, n (%)**			0.054 ^a^
Negative	36 (90.0)	37 (74.0)	
Positive	4 (10.0)	13 (26.0)	
**Brucella IgG, n (%)**			0.104 ^a^
Negative	9 (22.5)	5 (10.0)	
Positive	31 (77.5)	45 (90.0)	

WBC, white blood cell count; ALT, alanine aminotransferase; AST, aspartate aminotransferase; LDH, lactate dehydrogenase; GGT, gamma-glutamyl transferase; ALP, alkaline phosphatase; ESR, erythrocyte sedimentation rate; CRP, C-reactive protein; IgM, immunoglobulin M; IgG, immunoglobulin G. ^a^ Chi-square test; ^b^ Independent-samples *t* test; ^c^ Mann–Whitney U test. * Other occupations included civil servants, students, homemakers, retirees, and unemployed individuals.

**Table 6:** Univariable and multivariable logistic regression analyses for factors associated with focal organ involvement in patients with brucellosis.

	Univariable Analysis			Multivariable Analysis		
Variable	OR	95% CI	*p* value	Adjusted OR	95% CI	*p* value
Male sex	4.421	1.744–11.208	**0.002**			
Rural residence	3.273	1.289–8.310	**0.013**			
WBC count (per 100 cells/µL)	1.034	1.012–1.056	**0.003**			
Platelet count (per 1000 cells/µL)	0.992	0.988–0.999	**0.017**			
Lymphocyte count (/µL)	0.999	0.998–1.000	**0.016**			
LDH (U/L)	1.007	1.001–1.013	**0.024**			
ESR (mm/h)	1.045	1.012–1.080	**0.008**			
CRP (per 10 mg/L)	2.043	1.514–2.758	**<0.001**	1.662	1.202–2.298	**0.002**
Calprotectin (per 100 ng/mL)	3.738	2.234–6.255	**<0.001**	2.223	1.208–4.091	**0.010**
Prokineticin-2 (per 1 ng/mL)	3.328	1.883–5.881	**<0.001**	3.910	1.526–10.018	**0.005**
Hypertension	2.706	1.069–6.851	**0.036**			

OR, odds ratio; CI, confidence interval; WBC, white blood cell count; LDH, lactate dehydrogenase; ESR, erythrocyte sedimentation rate; CRP, C-reactive protein. Model fit: Hosmer–Lemeshow χ^2^ = 12.147, df = 8, *p* = 0.145. Multicollinearity assessment: VIF = 1.666 for CRP, 1.860 for PROK2, and 1.617 for calprotectin. For the univariable analyses, the reference categories for categorical variables were female sex, urban residence, and absence of hypertension. Continuous variables were modeled using the specified increments. For continuous biomarkers in the multivariable model, adjusted ORs represent the change in odds associated with a 1 ng/mL increase in PROK2, a 100 ng/mL increase in calprotectin, and a 10 mg/L increase in CRP.

**Figure 3:**
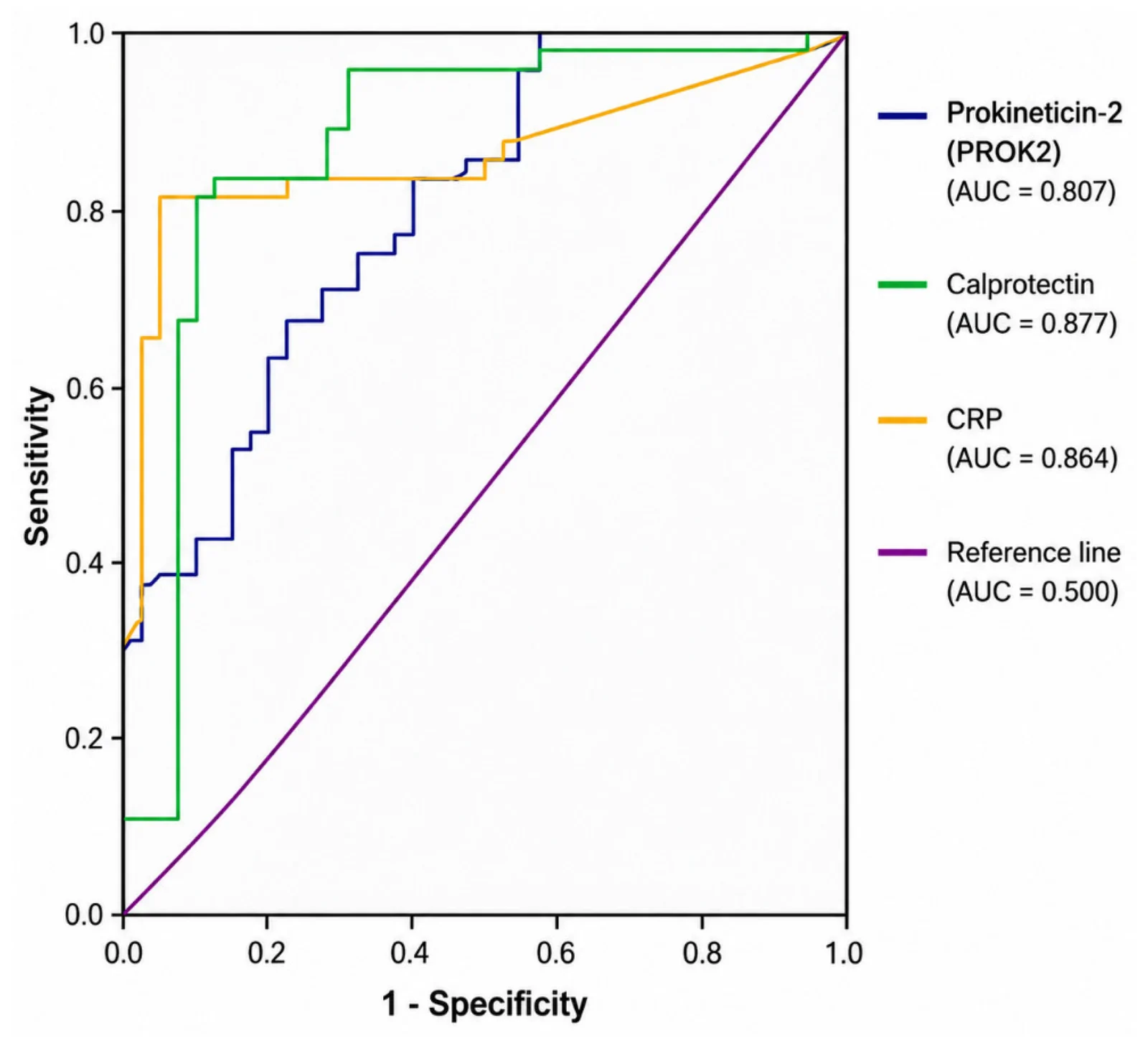
Receiver operating characteristic (ROC) curves illustrating the discriminative performance of serum prokineticin-2 (PROK2), calprotectin, and C-reactive protein (CRP) for distinguishing patients with and without focal organ involvement. The corresponding areas under the curve (AUCs), 95% confidence intervals, Youden-derived cut-off values, sensitivities, and specificities for the individual biomarkers are presented in Table 7.

In the ROC curve analysis, the AUC for serum calprotectin was 0.877 (95% CI: 0.794–0.959; *p* < 0.001). Corresponding AUC values were 0.864 (95% CI: 0.783–0.946; *p* < 0.001) for CRP and 0.807 (95% CI: 0.720–0.895; *p* < 0.001) for PROK2. At the Youden-derived exploratory cut-off of 127.25 ng/mL, serum calprotectin showed a sensitivity of 92.0% and a specificity of 70.0%. For serum CRP, the AUC was 0.864 (95% CI: 0.783–0.946; *p* < 0.001); at the exploratory Youden-derived cut-off of 14.07 mg/L, sensitivity was 82.0% and specificity was 77.5%. For serum PROK2, the AUC value was 0.807 (95% CI: 0.720–0.895; *p* < 0.001), and at the Youden-derived exploratory cut-off of 2.02 ng/mL, sensitivity was 82.0% and specificity was 60.0%. Because these cut-off values were derived and evaluated in the same cohort and were not subjected to internal or external validation, they should be regarded as exploratory and their observed performance may be optimistic. The results of the ROC analysis regarding the discriminative performance of serum biomarkers for focal organ involvement are presented in Table 7, and the ROC curves are shown in Figure 3.

## 4. Discussion

In this prospective case–control study, it was demonstrated that serum calprotectin and PROK2 levels were significantly higher in patients with brucellosis compared to the non-brucellosis control group. Furthermore, it was found that levels of both biomarkers were significantly higher in patients with focal organ involvement compared to those without focal organ involvement and that both biomarkers were independently associated with focal organ involvement in the multivariable analysis. These findings suggest that calprotectin and PROK2 are not merely biomarkers reflecting systemic inflammation but may also reflect biological processes associated with the clinical heterogeneity of brucellosis. To the best of our knowledge, this study is the first to evaluate serum PROK2 levels in human brucellosis and also contributes to the limited literature on circulating calprotectin levels in brucellosis.

Brucella species are facultative intracellular pathogens capable of surviving for long periods within host monocytes and macrophages, and control of the infection depends largely on the bactericidal activation of macrophages via an interferon-gamma (IFN-γ)-mediated Th1 response [10,30]. However, it is known that innate immunity—and particularly mediators derived from myeloid cells—plays a significant role in the early control of infection and the regulation of the inflammatory response [10,31]. In this context, it is thought that biomarkers reflecting the activation of neutrophils, monocytes, and macrophages may reflect not only the degree of systemic inflammation but also differences in the clinical phenotype of the disease. The known involvement of calprotectin and PROK2 in these cellular pathways provides a biological rationale for investigating their associations with inflammatory burden and focal organ involvement in brucellosis [32,33].

In our study, the finding that serum calprotectin levels were significantly higher in the brucellosis group compared to the control group supports the important role of the innate immune response in the pathogenesis of brucellosis. Calprotectin, an S100A8/S100A9 heterodimer primarily released by activated neutrophils and monocytes, is not only a sensitive marker of inflammatory burden but also a mediator involved in host defense mechanisms that limit microbial proliferation by binding metal ions and regulating leukocyte migration and the cytokine response [14,32,34]. Therefore, the elevated serum calprotectin levels observed in our study may reflect an increased inflammatory response; however, our data do not allow specific conclusions regarding the degree of myeloid cell activation. This interpretation is consistent with previous studies showing that calprotectin is associated with disease activity and clinical severity in various infectious and chronic inflammatory diseases [14,15,32]. Given the intracellular persistence of Brucella species, the observed increase in serum calprotectin may be related to the host inflammatory response; however, our findings do not establish a specific mechanism linking calprotectin to bacterial persistence. However, this potential mechanism requires validation through experimental and translational studies. Our findings suggest that similar biological mechanisms may also apply to human brucellosis and that calprotectin may be a promising biomarker for assessing the host inflammatory response.

One of the notable findings of our study is that serum PROK2 levels show significant differences across disease stages, with the highest levels detected in cases of acute brucellosis. PROK2 (Bv8) is a chemokine-like peptide expressed in inflammatory myeloid cells, primarily neutrophils, that regulates cell chemotaxis and cytokine release [33,35]. Experimental studies have shown that PROK2 expression in inflammatory tissues increases in parallel with granulocyte infiltration [32]. Therefore, the higher PROK2 levels observed in the acute phase may be associated with differences in the inflammatory response during the early stages of brucellosis; however, our data do not establish a specific underlying cellular mechanism. However, experimental and longitudinal studies evaluating PROK2 expression at different disease stages are needed to confirm these potential mechanisms.

The stage-dependent variation in PROK2 suggests that this biomarker may behave differently depending on the nature of the immune response in various types of infections. Indeed, it has been reported that circulating PROK2 levels decrease in sepsis and septic shock, and that lower PROK2 levels are found in deceased patients [36]. Although this observation is based primarily on experimental models and relatively limited clinical data, it indicates that the PROK2 response may be shaped in different ways depending on the type and severity of the infection. In contrast, brucellosis—unlike sepsis—is an infection that predominantly follows an intracellular persistent course, and in which the cellular immune response plays a crucial role in disease control [10,31]. The higher PROK2 levels observed in acute brucellosis may hypothetically be related to differences in myeloid-cell-associated inflammatory responses during the early stage of infection. Likewise, the variation in PROK2 levels across disease stages may reflect changes in the host inflammatory response; however, these interpretations remain speculative because cellular, mechanistic, and longitudinal measurements were not performed. This pattern suggests that PROK2 may vary according to disease stage; however, the present study does not allow conclusions regarding the specific biological mechanisms underlying this variation. Importantly, this stage-related pattern persisted after adjustment for age, hemoglobin, and CRP. However, the adjusted analysis showed a significant difference only between the acute and subacute groups, whereas the acute–chronic and subacute–chronic comparisons were not statistically significant. Therefore, PROK2 should not be interpreted as a validated marker for discriminating all disease stages, but rather as a biomarker showing stage-related variation that warrants further investigation.

The fact that both calprotectin and PROK2 levels were found to be significantly higher in patients with focal organ involvement, and that in multivariate analysis both biomarkers, along with CRP, were independently associated with focal involvement, Notably, while calprotectin levels did not show significant differences across disease stages in the adjusted analysis, they were significantly higher in patients with focal organ involvement than in those without focal organ involvement. Consistent with this finding, the adjusted disease-stage analysis showed no significant overall association between calprotectin levels and disease stage (*p* = 0.180), and none of the Bonferroni-adjusted pairwise comparisons reached statistical significance. These findings do not support calprotectin as a marker for distinguishing acute, subacute, and chronic brucellosis in the present cohort. This distinction suggests that calprotectin may be more closely associated with the inflammatory profile accompanying focal involvement than with the chronological stage of the disease. Various studies have shown that focal complications in brucellosis—including osteoarticular and genitourinary involvement—may be associated with a more pronounced inflammatory response, and that traditional inflammatory markers such as CRP and ESR may be elevated in patients with focal involvement [7,8,26,27]. However, it has been reported that the performance of these markers in distinguishing focal complications is limited when used alone [8,27]. The observed AUC values for calprotectin, CRP, and PROK2 indicate varying degrees of discrimination between patients with and without focal organ involvement in this cohort. These findings should be considered exploratory and require validation in independent populations. At the Youden-derived cut-off, calprotectin showed high sensitivity but moderate specificity; however, because this threshold was derived and evaluated in the same cohort without internal or external validation, its clinical applicability remains uncertain.

The higher rate of positive blood cultures in patients with focal organ involvement may reflect differences in the clinical phenotype of these patients. Clinical and laboratory characteristics associated with focal involvement and bacteremia have also been reported in previous patient series [7,26,37]. The higher calprotectin and PROK2 levels observed in patients with focal organ involvement suggest that these biomarkers are associated with differences in the inflammatory profile of these patients. However, for these biomarkers and the proposed cut-off values to be incorporated into routine clinical practice, they must be supported by external validation studies in different populations. Because these cut-off values were derived from the same study cohort using the Youden index, their diagnostic performance may be optimistically estimated. No internal or external validation was performed. Therefore, the reported cut-off values should be regarded as exploratory findings rather than clinically validated thresholds and require confirmation in independent external cohorts before any clinical application.

Some limitations of this study should be considered. First, the single-center design and relatively small sample size may have limited the statistical power of subgroup analyses, particularly regarding disease stage and focal organ involvement. In addition, because the study was conducted at a single tertiary referral center, referral and selection bias cannot be excluded, and patients with focal or more complex manifestations of brucellosis may have been disproportionately represented compared with the broader brucellosis population. The relatively wide confidence interval of the adjusted odds ratio for PROK2 (OR: 3.910; 95% CI: 1.526–10.018) in the multivariable model indicates that the precision of the effect estimate remains limited and warrants confirmation in larger cohorts. Second, since biomarker measurements were taken at a single time point at the time of diagnosis, it was not possible to assess the dynamic changes in calprotectin and PROK2 levels over the course of treatment response or disease progression. Furthermore, due to the observational nature of the study, the identified associations should not be interpreted causally. Because multiple secondary comparisons were performed across disease stages, clinical variables, and biomarkers without a global multiplicity adjustment, some nominally significant findings, particularly those with borderline *p* values, should be interpreted cautiously. The control group was clinically heterogeneous and included bacterial, viral, and acute non-infectious inflammatory conditions, each of which may differentially influence circulating inflammatory biomarker levels. This heterogeneity, including the presence of undiagnosed febrile illness and acute non-infectious inflammatory conditions, may have influenced between-group biomarker differences and should therefore be considered when interpreting the observed elevations in PROK2 and calprotectin. The heterogeneous composition of the symptomatic control group may also introduce spectrum bias, and the observed biomarker performance may therefore differ in other clinical populations with different distributions of febrile, infectious, or inflammatory conditions. The use of a heterogeneous symptomatic non-brucellosis control group represents a design characteristic that should be considered when interpreting the between-group biomarker differences and the generalizability of the findings. Because the diagnosis of brucellosis in this cohort was based predominantly on compatible clinical findings together with serological evidence, with microbiological confirmation available in only a minority of patients, the observed biomarker differences should be interpreted within the context of this case definition. In addition, disease-stage comparisons were performed among serologically defined cases classified according to symptom duration. Therefore, the limited microbiological confirmation across stage groups should be considered when interpreting stage-related biomarker differences. The absence of a healthy control group limits comparison with baseline biomarker levels in individuals without infection or inflammation. In addition, the absence of a healthy control group limits interpretation of the magnitude of biomarker elevation relative to baseline levels in individuals without infection or inflammation. Accordingly, our findings should be interpreted as demonstrating differences between patients with brucellosis and clinically relevant non-brucellosis controls, rather than as evidence of brucellosis-specific biomarker elevation relative to healthy individuals. The exclusion of patients with several chronic inflammatory, immunocompromising, and other comorbid conditions that could independently influence circulating PROK2 or calprotectin levels may also limit the generalizability of the observed biomarker performance to the broader population of patients evaluated for suspected brucellosis. Finally, the generalizability of our findings may be limited due to the single-center design, and the results need to be validated in larger, multicenter cohorts encompassing different geographic regions.

## 5. Conclusions

Serum PROK2 showed stage-related variation, with significantly higher adjusted levels in acute than in subacute brucellosis, while the acute–chronic and subacute–chronic comparisons were not significant. These findings indicate stage-related variation in PROK2 but do not establish reliable discrimination among acute, subacute, and chronic brucellosis. Both PROK2 and calprotectin were independently associated with focal organ involvement. Further multicenter and longitudinal studies are required to validate these findings and determine their clinical and prognostic value in the assessment of focal organ involvement.

## Figures and Tables

**Table 1:** Comparison of demographic, clinical, and laboratory characteristics between patients with brucellosis and non-brucellosis controls.

Parameters	Non-Brucellosis Controls (n = 60)	Patients with Brucellosis (n = 90)	*p* Value
**Demographic characteristics**			
Age (years), median (IQR)	47 [43–51.5]	48 [40–55]	0.213 ^c^
Sex, n (%)			0.721 ^a^
Female	19 (31.6)	31 (34.4)	
Male	41 (68.4)	59 (65.6)	
**Clinical characteristics**			
Fever, n (%)	33 (55.0)	45 (50.0)	0.548 ^a^
**Comorbidities**			
Hypertension, n (%)	26 (43.3)	31 (34.4)	0.272 ^a^
Diabetes mellitus, n (%)	9 (15.0)	15 (16.7)	0.925 ^a^
Congestive heart failure, n (%)	2 (3.3)	3 (3.3)	1.000 ^b^
Coronary artery disease, n (%)	13 (21.7)	18 (20.0)	0.805 ^a^
**Laboratory findings**			
WBC (/µL), median (IQR)	6710 [5295–8030]	6890 [5680–8850]	0.151
Hemoglobin (g/dL), mean ± SD	14.4 ± 1.7	14.7 ± 1.6	0.327 ^d^
Platelet count (/µL), mean ± SD	272,883 ± 64,549	253,144 ± 69,990	0.083 ^d^
Neutrophil count (/µL), median (IQR)	3710 [2745–5510]	3785 [2780–4940]	0.995 ^c^
Lymphocyte count (/µL), median (IQR)	2015 [1670–2380]	2020 [1590–2500]	0.890 ^c^
Creatinine (mg/dL), mean ± SD	0.7 ± 0.1	0.7 ± 0.1	0.214 ^d^
ESR (mm/h), median (IQR)	10 [4–21]	10.5 [3–24]	0.828 ^c^
CRP (mg/L), median (IQR)	3.7 [3.1–33.7]	19 [3.1–63.3]	**0.001 ^c^**
Prokineticin-2 (ng/mL), median (IQR)	2.00 [1.04–2.72]	2.51 [1.57–3.31]	**0.004 ^c^**
Calprotectin (ng/mL), median (IQR)	91.6 [62.9–142]	207.9 [71–334.5]	**0.001 ^c^**

WBC, white blood cell count; ESR, erythrocyte sedimentation rate; CRP, C-reactive protein; IQR, interquartile range; SD, standard deviation. ^a^ Chi-square test; ^b^ Fisher’s exact test; ^c^ Mann–Whitney U test; ^d^ independent-samples *t*-test.

**Table 2:** Comparison of demographic, clinical, laboratory, and serological characteristics according to disease stage.

Variable	Acute (n = 30)	Subacute (n = 30)	Chronic (n = 30)	*p* Value	Non-Brucellosis Controls (n = 60)
**Demographic characteristics**					
Age (years), mean ± SD	41.2 ± 11.2	47.2 ± 13.0	53.5 ± 10.3	**<0.001 ^a^**	47 [43–51.5]
Sex, n (%)				0.674 ^c^	
Female	9 (30.0)	10 (33.3)	12 (40.0)		19 (31.6)
Male	21 (70.0)	20 (66.7)	18 (60.0)		41 (68.4)
**Clinical characteristics**					
Fever, n (%)	12 (40.0)	13 (43.3)	20 (66.7)	0.079 ^c^	33 (55.0)
Weight loss, n (%)	6 (20.0)	7 (23.3)	14 (46.7)	**0.049 ^c^**	—
Loss of appetite, n (%)	16 (53.3)	19 (63.3)	24 (80.0)	0.090 ^c^	—
Sweating, n (%)	19 (63.3)	19 (63.3)	30 (100.0)	**0.001 ^c^**	—
Gastrointestinal symptoms, n (%)	15 (50.0)	6 (20.0)	3 (10.0)	**0.001 ^c^**	—
Fatigue, n (%)	28 (93.3)	28 (93.3)	23 (76.7)	0.100 ^d^	—
Depression, n (%)	0 (0.0)	2 (6.7)	1 (3.3)	0.770 ^d^	—
Arthralgia, n (%)	24 (80.0)	22 (73.3)	20 (66.7)	0.506 ^c^	—
Skin rash, n (%)	5 (16.7)	7 (23.3)	3 (10.0)	0.383 ^c^	—
Headache, n (%)	5 (16.7)	8 (26.7)	12 (40.0)	0.129 ^c^	—
Low back pain, n (%)	18 (60.0)	10 (33.3)	22 (73.3)	**0.006 ^c^**	—
**Focal organ involvement**					
Peripheral arthritis, n (%)	8 (26.7)	7 (23.3)	11 (36.7)	0.495 ^c^	—
Spondylodiscitis/Sacroiliitis, n (%)	7 (23.3)	6 (20.0)	9 (30.0)	0.656 ^c^	—
Hepatitis, n (%)	2 (6.7)	1 (3.3)	2 (6.7)	1.000 ^d^	—
Orchitis, n (%)	2 (6.7)	6 (20.0)	2 (6.7)	0.205 ^d^	—
Central nervous system involvement, n (%)	0 (0.0)	1 (3.3)	0 (0.0)	1.000 ^d^	—
Pneumonia, n (%)	0 (0.0)	1 (3.3)	0 (0.0)	1.000 ^d^	—
**Physical examination findings**					
Hepatomegaly, n (%)	5 (16.7)	6 (20.0)	8 (26.7)	0.627 ^c^	—
Splenomegaly, n (%)	4 (13.3)	2 (6.7)	7 (23.3)	0.217 ^d^	—
Lymphadenopathy, n (%)	2 (6.7)	0 (0.0)	3 (10.0)	0.363 ^d^	—
**Comorbidities**					
Hypertension, n (%)	6 (20.0)	12 (40.0)	13 (43.3)	0.121 ^c^	26 (43.3)
Diabetes mellitus, n (%)	4 (13.3)	5 (16.7)	6 (20.0)	0.413 ^d^	9 (15.0)
Congestive heart failure, n (%)	2 (6.7)	0 (0.0)	1 (3.3)	0.770 ^d^	2 (3.3)
Coronary artery disease, n (%)	4 (13.3)	8 (26.7)	6 (20.0)	0.435 ^c^	13 (21.7)
**Laboratory findings**					
WBC (/µL), median (IQR)	8225 (6380–9680)	8400 (5420–9650)	6050 (5160–6860)	**<0.001 ^b^**	6710 [5295–8030]
Hemoglobin (g/dL), mean ± SD	14.7 ± 1.3	14.1 ± 1.8	15.2 ± 1.4	**0.019 ^a^**	14.4 ± 1.7
Platelet count (/µL), mean ± SD	246,566.7 ± 67,956.2	258,400.0 ± 67889.2	254,466.7 ± 75707.6	0.804 ^a^	272,883 ± 64,549
Neutrophil count (/µL), median (IQR)	3580 (2840–5510)	4525 (3160–5470)	3595 (2630–4260)	0.070 ^b^	3710 [2745–5510]
Lymphocyte count (/µL), mean ± SD	1895.3 ± 479.2	2438.3 ± 800.9	1937.3 ± 549.3	**0.002 ^a^**	2015 [1670–2380]
Creatinine (mg/dL), mean ± SD	0.84 ± 0.16	0.78 ± 0.13	0.77 ± 0.13	0.107 ^a^	0.7 ± 0.1
Albumin (g/L), median (IQR)	42 (41–45)	42 (41–44)	43 (41–45)	0.568 ^b^	—
ALT (U/L), median (IQR)	33.5 (20–79)	34 (24–54)	28 (20–32)	0.232 ^b^	—
AST (U/L), median (IQR)	23 (17–55)	30 (18–41)	18.5 (14–24)	0.054 ^b^	—
LDH (U/L), median (IQR)	231 (185–320)	192 (152–245)	207 (178–240)	0.121 ^b^	—
GGT (U/L), median (IQR)	38 (18–65)	28.5 (21–62)	27 (19–61)	0.748 ^b^	—
ALP (U/L), median (IQR)	92 (69–123)	77 (41–95)	85 (64–88)	0.112 ^b^	—
ESR (mm/h), median (IQR)	4 (2–11)	11 (4–35)	15.5 (9–25)	**0.001 ^b^**	10 [4–21]
Ferritin (ng/mL), median (IQR)	98 (48–144)	78 (43–122)	70.5 (50–96)	0.782 ^b^	
CRP (mg/L), median (IQR)	33.10 (3.13–63.13)	52.82 (12.00–83.06)	9.64 (3.13–53.05)	0.027 ^b^	3.7 [3.1–33.7]
Prokineticin-2 (ng/mL), median (IQR)	3.17 (2.27–5.13)	2.25 (1.62–2.87)	2.07 (1.37–2.89)	**0.015 ^b^**	2.00 [1.04–2.72]
Calprotectin (ng/mL), median (IQR)	235.3 (62.8–330.1)	176.2 (85.2–336.5)	198.1 (76.5–336.5)	0.870 ^b^	91.6 [62.9–142]
**Serological findings**					
IgG positivity, n (%)	24 (80.0)	28 (93.3)	24 (80.0)	0.289 ^d^	—
IgM positivity, n (%)	12 (40.0)	3 (10)	2 (6.7)	**0.001 ^c^**	—
Coombs titer, n (%)				**0.030 ^d^**	—
1/160	11 (36.7)	2 (6.7)	5 (16.7)		
1/320	5 (16.7)	9 (30.0)	14 (46.7)		
1/640	14 (46.7)	19 (63.3)	11 (36.7)		
STA titer, n (%)				0.097 ^d^	—
Negative	13 (43.3)	14 (46.6)	13 (43.3)		
1/20	1 (3.3)	5 (16.6)	10 (33.3)		
1/40	0 (0.0)	1 (3.3)	0 (0.0)		
1/80	5 (16.6)	5 (16.6)	5 (16.6)		
1/160	1 (3.3)	2 (6.6)	0 (0.0)		
1/320	5 (16.6)	2 (6.6)	1 (3.3)		
1/640	3 (10.0)	1 (3.3)	1 (3.3)		
≥1/1280	2 (6.6)	0 (0.0)	0 (0.0)		
Rose Bengal positivity, n (%)	25 (83.3)	25 (83.3)	22 (73.3)	0.535	—
Blood culture positivity, n (%)	3 (10.0)	1 (3.3)	4 (13.3)	0.522	—

Continuous variables are presented as mean ± standard deviation or median (interquartile range [IQR]), as appropriate. HT, hypertension; DM, diabetes mellitus; CHF, congestive heart failure; CAD, coronary artery disease; WBC, white blood cell count; ALT, alanine aminotransferase; AST, aspartate aminotransferase; LDH, lactate dehydrogenase; GGT, gamma-glutamyl transferase; ALP, alkaline phosphatase; ESR, erythrocyte sedimentation rate; CRP, C-reactive protein; IgG, immunoglobulin G; IgM, immunoglobulin M; STA, standard tube agglutination test; IQR, interquartile range; SD, standard deviation. ^a^ One-way analysis of variance (ANOVA); ^b^ Kruskal–Wallis H test; ^c^ Chi-square test; ^d^ Fisher’s exact test. *p* values refer exclusively to comparisons among the acute, subacute, and chronic brucellosis groups; the non-brucellosis control group is presented for descriptive comparison only and was not included in these statistical tests.

**Table 3:** Bonferroni-adjusted pairwise comparisons of demographic, clinical, and laboratory variables among the acute, subacute, and chronic groups.

Variable	Acute vs. Subacute	Acute vs. Chronic	Subacute vs. Chronic
**Demographic**			
Age ^a^	0.120	**<0.001**	0.088
**Clinical characteristics**			
Sweating ^d^	1.000	**<0.001**	**<0.001**
Gastrointestinal symptoms ^d^	**0.015**	**0.001**	0.478
Lower-back pain ^d^	**0.038**	0.273	**0.002**
**Laboratory findings**			
WBC ^c^	0.520	**<0.001**	**0.003**
Hemoglobin ^a^	0.249	0.417	**0.014**
Lymphocyte ^b^	**0.003**	0.963	**0.007**
Platelet ^a^	0.793	0.902	0.975
Creatinine ^a^	0.211	0.124	0.959
CRP ^c^	0.076	0.381	**0.009**
ESR ^c^	0.520	**0.022**	**<0.001**
Prokineticin-2 ^c^	**0.012**	**0.014**	0.615

Data are presented as pairwise comparisons with Bonferroni-adjusted *p* values. WBC, white blood cell count; CRP, C-reactive protein; ESR, erythrocyte sedimentation rate. ^a^ Tukey’s post hoc test; ^b^ Games–Howell post hoc test; ^c^ pairwise Mann–Whitney U test with Bonferroni correction; ^d^ Pairwise Pearson chi-square or Fisher’s exact test with Bonferroni correction, as appropriate.

**Table 7:** Discriminative performance of serum biomarkers for focal organ involvement in patients with brucellosis.

Marker	AUC (95% CI)	*p* Value	Youden-Derived Cut-Off	Sensitivity (%)	Specificity (%)
Calprotectin (ng/mL)	0.877 (0.794–0.959)	<0.001	127.25	92.0	70.0
CRP (mg/L)	0.864 (0.783–0.946)	<0.001	14.07	82.0	77.5
Prokineticin-2 (ng/mL)	0.807 (0.720–0.895)	<0.001	2.02	82.0	60.0

AUC, area under the curve; CI, confidence interval; CRP, C-reactive protein; ROC, receiver operating characteristic.

## Data Availability

The data presented in this study are available from the corresponding author upon reasonable request.
